# Electric Susceptibility at Partial Coverage of a Circular One-Side Access Capacitive Sensor with Rigid Polyurethane Foams

**DOI:** 10.3390/s24103003

**Published:** 2024-05-09

**Authors:** Ilze Beverte

**Affiliations:** Institute for Mechanics of Materials, University of Latvia, LV-1004 Riga, Latvia; ilze.beverte@lu.lv; Tel.: +371-29-46-42-50

**Keywords:** capacitive sensor, one-side access, circular symmetry, polyurethane foams, partial coverage, permittivity, electric susceptibility, surface charge

## Abstract

The capability of dielectric measurements was significantly increased with the development of capacitive one-side access physical sensors. Complete samples give no opportunity to study electric susceptibility at a partial coverage of the one-side access sensor’s active area; therefore, partial samples are proposed. The electric susceptibility at the partial coverage of a circular one-side access sensor with cylinders and shells is investigated for polyurethane materials. The implementation of the relative partial susceptibility permitted us to transform the calculated susceptibility data to a common scale of 0.0–1.0 and to outline the main trends for PU materials. The partial susceptibility, relative partial susceptibility, and change rate of relative partial susceptibility exhibited dependence on the coverage coefficient of the sensor’s active area. The overall character of the curves for the change rate of the relative partial susceptibility, characterised by slopes of lines and the ratio of the change rate in the centre and near the gap, corresponds with the character of the surface charge density distribution curves, calculated from mathematical models. The elaborated methods may be useful in the design and optimization of capacitive OSA sensors of other configurations of electrodes, independent of the particular technical solution.

## 1. Introduction

In the non-destructive evaluation of dielectric materials, in the frequency band up to 10 MHz, the capacitance method is one of the main testing methods [1]. The capability of dielectric measurements was significantly increased with the development of capacitive one-side access (OSA) physical sensors, which permitted non-destructive testing and the characterization of materials without preparation of specimens [2,3,4,5], by merely pushing the sensor against one side of the test object. Several problems exist when working with capacitive OSA sensors: they excite test objects with a non-homogeneous electric field, have low sensing (geometric) capacitances, and high stray capacitances; bulk reference specimens are needed, whose minimum thickness (height) is limited by the effective depth of the penetration of the electrical field. Contrary to the parallel-plate capacitive sensors, working with a homogeneous electric field, there are no simple formulas for calculating the sensing capacities of OSA sensors at a complete or partial coverage with a dielectric [3,6,7].

The intensity maximum of a low-frequency excitation field is situated in the direct vicinity of the active area of a capacitive OSA transducer’s electrodes [2]. The penetration depth of the excitation field into the test object determines the lateral dimensions (height/thickness) appropriate for permittivity measurements [2,3,7,8]. In [7], the penetration depth of a concentric, coplanar, and capacitive OSA sensor was estimated by identifying the test object’s height, for which the capacitance is 3% or 10% smaller than its value when in contact with a similar, but infinitely high, test-piece. For a given sensor configuration, the sensor penetration depth increases as the test object’s permittivity increases. The measured permittivity of polyurethane foams and PTFE was investigated in [8] with a dependence on the OSA sensor’s working volume, when the latter is filled partly along the lateral dimension (height) of the samples. The height of the samples was gradually decreased, and a 3% criterion to detect the penetration depth was applied.

Mathematical modelling and numerical calculations in [7] outlined an optimal two-electrode OSA sensor configuration for a given width of the gap, which ensured the maximum transcapacitance. Utilizing the spatial domain Green’s functions and the method of moments [9,10], the sensor surface charge density was calculated, and then the sensor’s transcapacitance was determined from its surface charge. In [11], the charge density on circular electrodes of a two-electrode OSA sensor was assumed to be constant, and the average potential of the electrodes was calculated. In [12,13], a circular three-electrode OSA sensor is considered. A jump in the normal component of the electric field across an interface is defined, which would be equal to the surface charge density in the absence of ohmic conduction in the medium. The jump is zero at every interface except in the plane of the electrodes. For every Fourier–Bessel mode [14], the jump is related to the difference in the surface capacitance density.

The regions of an OSA sensor that contribute most to the sensor’s capacitance are the outer edge of the inner electrode and the inner edge of the outer electrode, where the surface charge density is the highest [6,7,15]. This results in a low capacitance of the sensor. The most sensitive zone of the sensor closely corresponds to the location of the gap between its two electrodes, and for sensors with a relatively large radius of the inner electrode, there will be an insensitive zone at the centre [6]. Empirical knowledge of a certain physical property of dielectric material, dependent on the surface charge distribution on the active area, would facilitate a performance evaluation, modelling, and the design of optimal proportions of electrodes’ dimensions [16,17,18,19,20,21,22].

PU foam is a “Polymer-gas” composite, which has the same matrix—monolithic polyurethane, in the whole range of densities, i.e., 30–1280 kg/m^3^ [23,24,25]. For light-weight PU foams (density of 30 kg/m^3^), permittivity ε ≈ 1.065, for medium weight (550 kg/m^3^), ε ≈ 2.10, and for monolithic polyurethane (1280 kg/m^3^), ε ≈ 3.35 (all at a frequency of 1 kHz) [8,26,27]. Rigid polyurethane (PU) foams [23,24] exhibit low dielectric interference and nearly non-dispersive permittivity; therefore, excellent dielectric performance can be ensured in a wide frequency range. The foams are comparatively easy to process mechanically, which makes them appropriate for a study of permittivity and electric susceptibility at a partial coverage of an OSA sensor.

A complete dielectric sample covers the total active area of the OSA sensor, and the permittivity, measured for it, is the true permittivity, which characterises the overall effect of the electric charge on the sample. Complete samples provide no opportunity to study permittivity and electric susceptibility at a partial coverage of the active area and to estimate their correspondence to the charge density distribution. Therefore, partial samples are proposed for measurements of permittivity and the study of electric susceptibility. This study aims to investigate electric susceptibility at a partial coverage of a circular capacitive OSA physical sensor with samples—cylinders and shells, made from PU foams and monolithic polyurethanes. A transition to the relative partial electric susceptibility permitted us to transform the calculated data of the partial susceptibility to a common scale of 0.0–1.0 and to outline the main trends for PU materials of significantly differing true permittivity. The change rate of the relative partial susceptibility with a dependence on the coverage coefficients of the active area and the subsample’s radius is evaluated. A comparison with the results of other authors is made.

## 2. Materials and Methods

### 2.1. Polyurethane Materials

Rigid closed-cell petrochemical PU foams were made at apparent core densities of 30 kg/m^3^ < ρ < 80 kg/m^3^, in blocks, according to the formulation in [8,26,27]. The apparent core density (density) was determined in correspondence with ISO 845:2006 [28]. The difference in densities was achieved by varying the amount of physical or chemical blowing agents. To make monolithic polyurethane, a liquid mixture of the same formulation as for PU foams was poured into polyethylene ampoules with an inner diameter of 25.4 mm and a length of 115 mm; no foaming agent was added. The ampoules were centrifuged for ~20 min at 5000 rpm in a centrifuge Sigma 3-30KS (Sigma Laborzentrifugen GmbH, Osterode am Harz, Germany) [26]. The technology ensured that ≈2/3 of the length of the cylindrical part of the PU rods was free from gaseous inclusions.

Industrially manufactured rigid petrochemical PU foam SikaBlock, with densities of 85 kg/m^3^ ≤ ρ ≤ 415 kg/m^3^, monolithic polyurethane SikaBlock-M945, and a comparative material—monolithic epoxy LAB 975 NEW—were acquired from Sika JSC (Baar, Switzerland).

Depending on the production technology, PU foams can exhibit anisotropy of structure and properties [29,30], when the permittivity and electric susceptibility ε_ij_ and χ_ij_ are the 2nd-rank tensors. Only isotropic PU foams are considered further.

### 2.2. Capacitive One-Side Access Sensor

**Figure 1:**
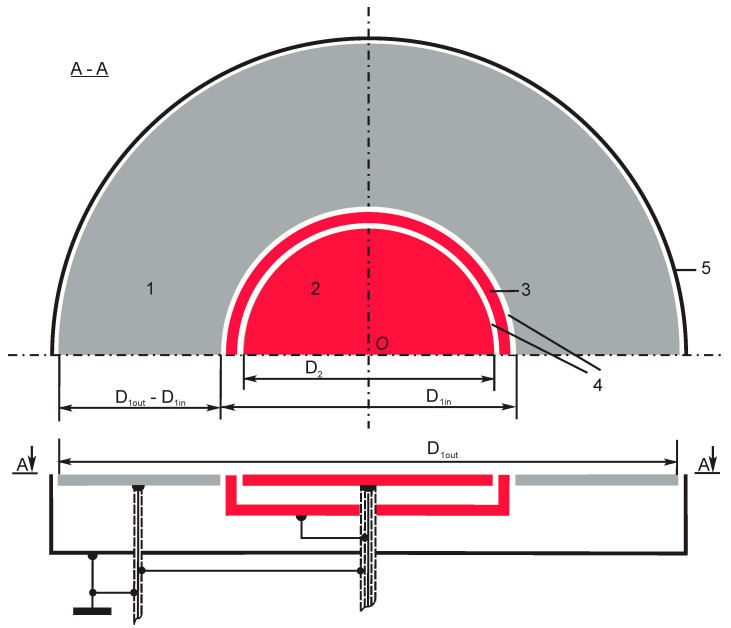
The electrodes: (1) Driven (D_1in_ = 21.0 mm, D_1out_ = 45.0 mm), (2) Sensing (diameter D_2_ = 16.8 mm), and (3) Guard (width of 1.00 mm) as well as (4) Insulator (width of 0.55 mm) and (5) Grounded screen of the OSA sensor.

Permittivity was measured with an experimental dielectric spectrometer equipped with a capacitive sensor of the one-side access (OSA) type [3,8,26,27]. The dielectric sample was placed on the active area of the sensor, Figure 1, and excited via electrodes, by an electrical field generated by sinusoidal voltage signals. The amplitude value of the sinusoidal excitation signals was U_0_ = 20 V. The signals were generated at discrete frequencies, increasing in a geometric progression as follows:

f_n_ = f_1_, 2f_1_, …, 2^(n − 1)^f_1_ Hz, where f_1_ = 10 Hz, n = 1, 2, …, 16, f = 10, 20, …, 327,680 Hz,(1)

where n is the ordeal number of a frequency. In the capacitive sensor system (CSS), the driven electrode №1 and sensing electrode №2 are connected to the multi-frequency excitation generator; the driven electrode is connected directly to the sensing electrode through a reference capacitor C_ref_ [3].

To carry out stray-immune capacitance measurements, the sensing electrode, the reference capacitor, and the unity gain buffer amplifier are covered with a screen, which forms the guard electrode №3 on the active area of CSS around the sensing electrode, as shown in Figure 1. The guard electrode is fed by the same voltage as the sensing electrode (active guarding) through a voltage follower, thus suppressing the electric field between the driven electrode and the sensing electrode outside the sensor’s active surface.

The accuracy of the spectrometer in permittivity measurements, in conditions of repeatability, was estimated with the expanded uncertainty U_S_95.45% = ±0.01 [8,31,32]. The calibration of the spectrometer was made before each measurement, with respect to permittivity, delivered by the sensor in the air. Three consecutive measurements of permittivity spectra were made for each data point.

### 2.3. Dielectric Losses

The dielectric loss part ε″(f) of the complex permittivity $\tilde{\varepsilon }$(jf) = ε′(f) − jε″(f) was evaluated for PU foams and monolithic polyurethane, using data from direct measurements and scientific information sources [8]. The dielectric losses of PU materials were measured with a broadband dielectric spectrometer BDS-50 (Novocontrol Technologies GmbH & Co. KG, Montabaur, Germany), comprising a parallel plate capacitor, for samples with a diameter of 30 mm and a thickness of 2 mm. Five measurements were made for each data point. The real and the imaginary parts ε′(f) and ε″(f) were compared.

### 2.4. Lateral Dimensions of a Complete Sample

In order to study the special cases of the partial coverage of the OSA sensor’s active area, several benchmark experiments were carried out (Points 2.4., 2.5., 2.6., and 2.8.), and their results were analysed (Points 4.2., 4.3., 4.4., and 4.6.).

Let us consider a PU foam sample with a diameter d = D_0_ = 45 mm and thickness t = 65 mm. The cross-sectional surface of the sample covers the whole active area of the circular OSA sensor, and the thickness considerably exceeds the penetration depth of the low-frequency electric field into the PU foams [1 d c, PTFE]. The impact of the lateral dimension (the thickness) of such a sample on the permittivity was studied. The intensity maximum of the dielectric spectrometer’s low-frequency excitation field is situated in the direct vicinity of the active area of the OSA sensor’s electrodes [2,8]. Therefore, first, the permittivity of the samples was determined. Then, a thin layer, thickness Δt = 0.5–2.0 mm, was cut from the top of the sample, and the permittivity of the remaining part was measured. Then, the next layer with a thickness Δt = 0.5–2.0 mm was cut, etc. The experimental data were plotted against the thickness values of the sample. Then the penetration depth of the electric field of a certain frequency f was defined as such a thickness t_3%_ of a sample, at which the electric susceptibility is 3% less than the true susceptibility χ_t_ = ε_t_ − 1.0 of an infinitely thick sample, χ = 97% χ_t_ [7,8].

A sample is considered complete if (1) its cross-sectional surface covers the whole active area of the sensor and (2) its thickness equals or exceeds the penetration depth of the electric field of a certain frequency into the given dielectric. A measurement on a complete sample provides the true permittivity ε_t_ of the dielectric material.

The reading of the dielectric spectrometer for any sample, which either (1) covers the active area of the sensor partially and/or (2) has a thickness, smaller than the penetration depth of the electric field of a certain frequency, is denoted further as “The measured permittivity ε_m_”.

### 2.5. Transversal Dimensions of Samples

**Figure 2:**
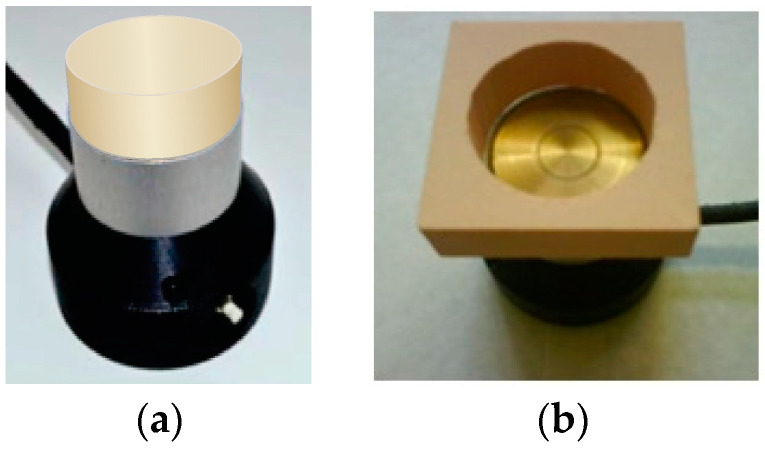
PU foam samples on the active area of the sensor: (**a**) A cylindrical complete sample with a diameter d = D_0_ = 45 mm and a thickness of 20 mm and (**b**) a parallelepiped with a concentric cylindrical hole.

The impact of the transversal dimensions of a sample on the permittivity was studied for parallelepiped-shaped samples of dimensions (1) 20 × 65 × 65 mm and (2) 20 × 45 × 45 mm and for (3) cylindrical samples with a diameter d = D_0_ = 45 mm and a thickness of 20 mm, Figure 2a. The “(2)” parallelepipeds were made from the “(1)” ones, and then the cylinders were made from the “(2)” parallelepipeds. All three kinds of samples are considered complete, since covering the whole active area is ensured. The permittivity of samples “(1)”, “(2)”, and “(3)” was determined for lab-made PU foam (50 kg/m^3^), PU foam SikaBlock-M150 (144 kg/m^3^), and SikaBlock-M450 (415 kg/m^3^).

Another experiment was performed for three other parallelepipeds of the kind “(1)” (20 × 65 × 65 mm), made from the same PU foams. First, the permittivity was measured for each parallelepiped, and then a concentric cylindrical hole, diameter d ≈ D_0_ = 45 mm, was made in each sample. The sample was placed on the isolated circular case of the sensor, Figure 2b, and a reading of the spectrometer was made.

### 2.6. Inner Vertical Surfaces

**Figure 3:**
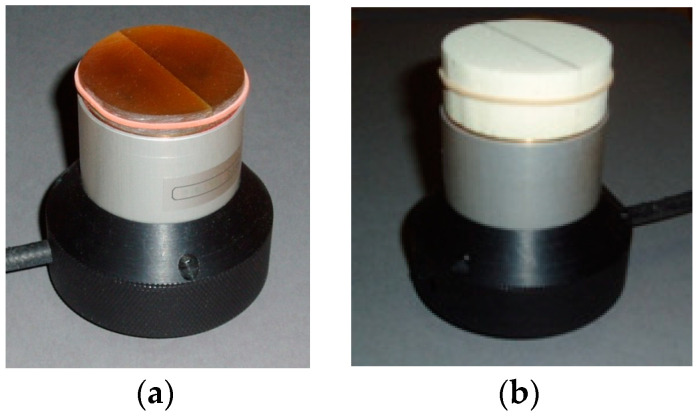
Complete samples with inner vertical surfaces on the active area of the sensor: (**a**) Lab-made monolithic PU and (**b**) PU foam SikaBlock-M450.

The impact of inner vertical surfaces in a complete, circular cylindrical sample on the measured permittivity was estimated experimentally. The task arises due to the limited transversal and lateral dimensions of the lab-made monolithic PU rods, when only one semi-cylinder (D_0_ = 45 mm and h = 12 mm) can be made from a PU rod. Side by side, the two semi-cylinders form a combined sample with two inner vertical surfaces along the diagonal. The semi-cylinders were pushed together, fixed firmly with an elastic band along the outer circular perimeter, Figure 3a, and the reading of the spectrometer was made.

The experiment was carried out also for a monolithic PU SikaBlock-M960. The dielectric SikaBlock-M960 was available in blocks of sufficiently large dimensions to make complete cylindrical samples of thickness t = 12 mm and diameter D_0_ = 45 mm. First, the permittivity of a complete, continuous sample was measured, and then it was cut into two semi-cylindrical parts. The parts were pushed together and fixed firmly with the elastic band, and the reading of the spectrometer was made. The experiment was made for the PU foam SikaBlock-M450 as well; the diameter of a sample was d_c_ = D_0_ = 45 mm, and the thickness was t = 25 mm, as shown in Figure 3b.

### 2.7. Coverage Coefficients and the Corresponding Cylinders and Shells

Cylinders and shells of circular symmetry were used to study the partial susceptibility of rigid PU foams and monolithic polyurethanes, depending on the coverage coefficients of the active area of the OSA capacitive sensor. When a cylinder or a shell is placed concentrically on the circular active area, its coverage can be characterised by coverage coefficients k_c_ and k_s_ as follows:

(2) $$k_{c}=\frac{S_{c}}{S_{0}}={\left(\frac{d_{c}}{D_{0}}\right)}^{2}={\left(\frac{r_{c}}{R_{0}}\right)}^{2},\;k_{s}=\frac{S_{s}}{S_{0}}=\frac{\pi \left(D_{0}^{2}-d_{sin}^{2}\right)}{\pi D_{0}^{2}}=1.0-{\left(\frac{d_{sin}}{D_{0}}\right)}^{2}=1.0-{\left(\frac{r_{sin}}{R_{0}}\right)}^{2},\;d_{sin}=d_{\mathrm{sout}}-t,\;0.0\leq r_{c},\;r_{\sin}\leq R_{0}\text{ and }0.0\leq k_{c},\;k_{s}\leq 1.0,$$

where d_c_—the diameter of the cylinder (d_c_ ≤ D_0_), t—the thickness of the shell wall, d_sin_ and d_sout_ = D_0_ = 45 mm—the inner and the outer diameters of the shell (d_sin_ ≤ d_sout_), S_c_—the cross-sectional area of a cylinder, S_s_—the cross-sectional area of a shell and the total active area of the sensor, S_0_ = π/4D_0_^2^ = 1590.4 mm^2^.

**Figure 4:**
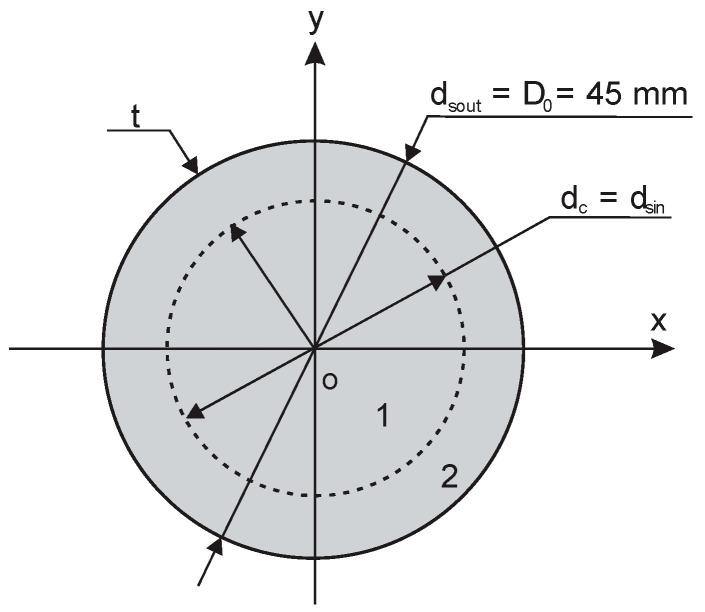
Cross-section of corresponding circular figures: cylinder 1 and shell 2.

A shell of outer diameter d_sout_ = D_0_ = 45 mm and inner diameter d_sin_ is denoted as corresponding to a cylinder of diameter d_c_, if d_sin_ = d_c_. The summary cross-sectional area of a cylinder and a corresponding shell equals the sensor’s active area, providing 100% coverage, Figure 4, as follows:

S_c_ + S_s_ = S_0_ and k_c_ + k_s_ = 1.0. (3)

The radius of a cylinder and the inner radius of a shell are related to the coverage coefficients k_c_ and k_s_ as follows:

(4) $$r_{c}=1/2D_{0}\sqrt{k_{c}}\;\mathrm{and}\;r_{\sin}=1/2D_{0}\sqrt{{1-k}_{sin}}.$$

The dependence of k_c_ and k_s_ and their change rates k_c_′ = $\frac{dk_{c}}{dr_{c}}=\frac{2r_{c}}{R_{0}^{2}}$ and k_s_′ = $\frac{{dk}_{s}}{{dr}_{sin}}=-2\frac{r_{sin}}{R_{0}^{2}}$ on the radial dimension of the subsamples was evaluated.

### 2.8. The Measured Permittivity of Concentric Circular Subsamples

**Figure 5:**
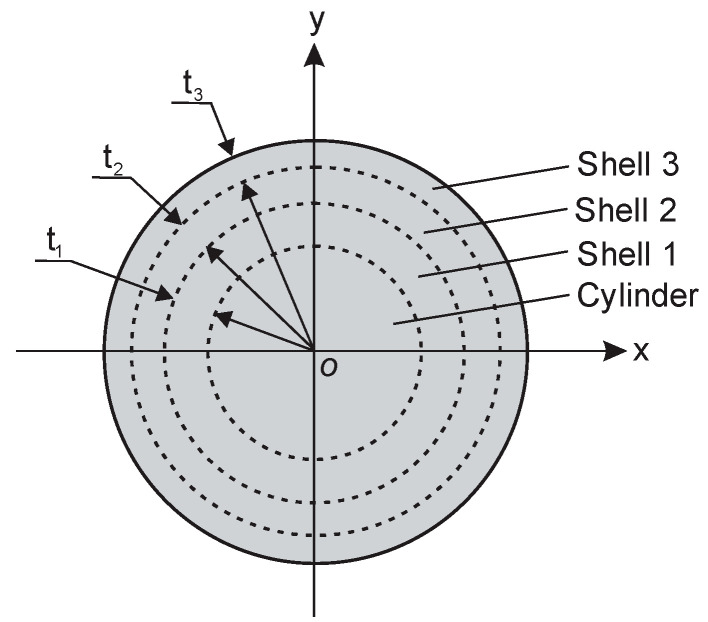
Cross-section of the concentric circular subsamples.

A complete cylindrical sample of PU foam SikaBlock-M240 (density of 230 kg/m^3^, thickness t = 20 mm, diameter d = D_0_ = 45 mm, and cross-sectional area S = S_0_ = 1590.4 mm^2^) was made, and its true permittivity was measured. The sample was cut into four concentric circular subsamples—a cylinder and three cylindrical shells, Figure 5. The dimensions of the subsamples were calculated to ensure an equal cross-sectional area S = S_0_/4 = 397.6 mm^2^, neglecting the finite width of the cutting lines. Then, the actual dimensions of the subsamples were measured, and the coverage coefficients were calculated. The measured permittivity ε_m_ was determined for each subsample. To estimate the impact of PU foams, lost as sawdust, the four subsamples were put one into another to form a combined, quasi-complete sample, and its measured permittivity ε_m_ was determined.

The true and the partial electric susceptibility χ_t_ and χ_p_ were calculated from experimental data of the true and the measured permittivity ε_t_ and ε_m_.

### 2.9. The Measured Permittivity at a Partial Coverage

**Figure 6:**
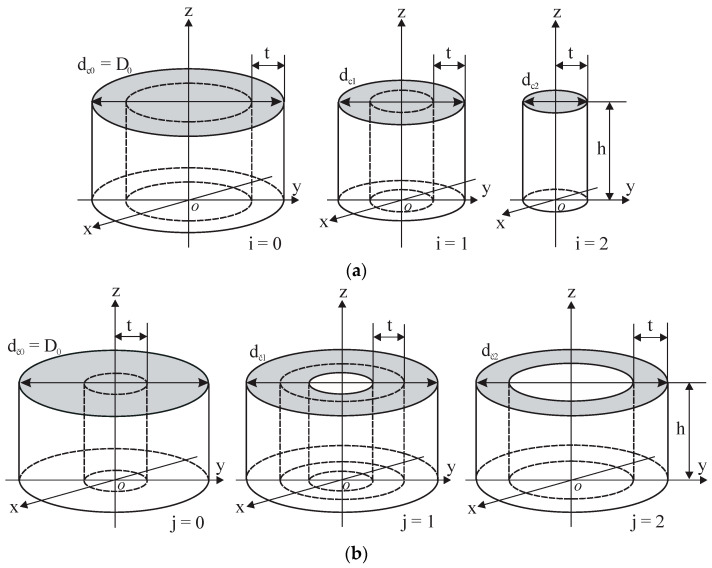
Complete samples (i = 0 and j = 0) and circular subsamples: (**a**) cylinders (i = 1 and 2) and (**b**) cylindrical shells (j = 1 and 2).

To study the partial electric susceptibility of rigid PU foams and monolithic polyurethanes, with a dependence on the coverage coefficients of the sensor, two identical right, circular, and cylindrical complete samples (i = 0 and j = 0, thickness t = 20 mm, and diameter D_0_ = 45 mm) were made from each dielectric material, as shown in Figure 6.

The true permittivity ε_t_ of both complete samples was measured. Then a concentric, circular, and cylindrical subsample (i = 1) was made by removing a shell of wall thickness t from outside of the first complete sample, as in Figure 6a, and the measured permittivity of the subsample was determined. The next subsample (i = 2) was made, and its measured permittivity was determined, etc. A concentric, circular, and cylindrical shell was made by removing a cylinder of radius t from the centre of the second complete sample, as in Figure 6b, and the measured permittivity of the subsample j = 1 was determined, etc. Then the next shell (j = 2) was made by removing a shell of wall thickness t from the centre of the shell j = 1, and the permittivity of the shell, j = 2, was measured, etc. Altogether, 5 … 6 subsamples were made from each complete sample.

The measured permittivity of lab-made PU foams (density 32 and 78 kg/m^3^) and industrial PU foam SikaBlock-M80 (85 kg/m^3^), SikaBlock-M150 (144 kg/m^3^), SikaBlock-M240 (226 kg/m^3^), and SikaBlock-M450 (415 kg/m^3^) was determined for cylinders. The measured permittivity of the shells was determined for light- to medium-weight PU foam SikaBlock-M80 (85 kg/m^3^), SikaBlock-M150 (144 kg/m^3^), and SikaBlock-M450 (415 kg/m^3^).

The measured permittivity of monolithic dielectrics was determined for the cylinders, made of the lab-made monolithic polyurethane (1280 kg/m^3^), the industrial monolithic polyurethane SikaBlock-M945 (1352 kg/m^3^), and epoxy Lab-975New (708 kg/m^3^). The shell-shaped subsamples of these high-density materials were not made due to technical challenges. The thickness of lab-made monolithic PU complete samples was t = 12 mm.

The spectrometer and the test materials were situated in the same premises for the entire study to attain thermodynamic equilibrium; no conditioning was made for the samples. The permittivity spectra were approximated according to methodology [8]. The true and the partial electric susceptibility, χ_t_ and χ_p_, were calculated from experimental data of the true and the measured permittivity values, ε_t_ and ε_m_, of the listed dielectric materials.

Data, corresponding to a frequency f = 1/2(f_7_ + f_8_) = 960 Hz ≈ 1 kHz, are given in this paper. For two density cases, (1) light-weight PU foam SikaBlock-M80 (85 kg/m^3^) and (2) Monolithic PU SikaBlock-M945 (1352 kg/m^3^) data, corresponding to f_13_ = 40,960 Hz, are given as well to evaluate the dielectric dispersion [26].

## 3. Theoretical

### 3.1. Model Functions

The true and the partial susceptibility of a dielectric material, χ_t_ and χ_p_, are related to the true and the measured permittivity, ε_t_ and ε_m_, as follows:

χ_t_ = ε_t_ − 1.0 and χ_p_ = ε_m_ − 1.0. (5)

The experimental data curves ε_m_ = ε_m_(r) of cylinders and shells were recalculated to χ_p_ = χ_p_(r), and the relative partial susceptibility RΧ was implemented by normalising the partial susceptibility χ_p_(r) with χ_t_:

(6) $$RX\left(r_{c}\right)=\frac{\chi_{pc}\left(r_{c}\right)}{\chi_{t}}=\frac{\varepsilon_{mc}\left(r_{c}\right)-1.0}{\varepsilon_{t}-1.0}\;\mathrm{and}\;RX\left(r_{sin}\right)=\frac{\chi_{ps}\left(r_{sin}\right)}{\chi_{t}}=\frac{\varepsilon_{ms}\left(r_{sin}\right)-1.0}{\varepsilon_{t}-1.0},$$

where r_c_ or r_sin_ are the radiuses of cylinders and shells. With such a definition, RX characterises the fraction, which a subsample’s partial susceptibility forms from the true susceptibility of a complete sample. The values of the relative partial susceptibility lie in a common scale of 0.0 ≤ RΧ(r_c_) and RΧ(r_sin_) ≤ 1.0. Analysis of calculated RΧ(r_c_) and RΧ(r_sin_) data curves identified an inflection point at r_e2_ < r_infl_ < r_e1in_, where r_e1in_ is the inner radius of the electrode №1 and r_e2_ is the radius of the electrode №2.

To model the calculated data curves RX(r_c_) and RΧ(r_sin_), different functions were tested, but none of them described the relationships properly. Therefore, the model function is proposed as a combination of two normalized functions: (1) a cumulative normal distribution and (2) a cumulative lognormal distribution. For cylinders, the model function Φ(r_c_) ≈ RX(r_c_) equals the following:

(7) $$f_{1}(r_{c})=\frac{1}{\sqrt{2\pi }\sigma_{1}}\int_{0}^{r_{c}}e^{-\frac{{\left(r-\mu_{1}\right)}^{2}}{2\sigma_{1}^{2}}}dr\text{ at }r_{c}<r_{\mathrm{infl}}\text{ and}\;f_{2}(r_{c})=\frac{1}{\sqrt{2\pi }\sigma_{2}r_{c}}\int_{r_{c}}^{R_{0}}e^{-\frac{{\left[ln(r)-\mu_{2}\right]}^{2}}{2\sigma_{2}^{2}}}dr\text{ at }r_{c}\geq r_{\mathrm{infl}},$$

where parameters μ_1_, μ_2_ are mean values and σ_1_, σ_2_ are standard deviations. The functions f_1_(r_c_) and f_2_(r_c_) are joined at the intersection point, which coincides with the inflection point. To join f_1_(r_c_) and f_2_(r_c_) smoothly, their average value can be assigned to RX at the joining point, when necessary.

### 3.2. Change Rate of the Relative Partial Susceptibility

In order to characterise the change rate of the relative partial susceptibility RX, with a dependence on the radius of dielectric cylinders, ΔRX(r_c_)/Δr_c_ can be estimated from the model function Φ(r_c_) as follows:

ΔRX(r_c_)/Δr_c_ ≈ ΔΦ(r_c_)/Δr_c_. (8)

But, with an increase in the cylinder’s radius r_c_, the coverage coefficient k_c_ increases nonlinearly, as in Equation (2), and the increments of the coverage coefficient, which correspond to constant increments of radius Δr_c_ = const., are radius-dependent:

(9) $${k_{c}}^{\prime }\approx \frac{\Delta k_{c}}{\Delta r_{c}}=\frac{2r_{c}}{R_{0}^{2}}\;\mathrm{and}\;\Delta k_{c}=2r_{c}\frac{∆r_{c}}{R_{0}^{2}}.$$

Therefore, estimation (8) is not fit for the purpose, and the change rate of RX has to be determined at constant increments of the OSA sensor’s coverage. Substituting $r_{c}=R_{0}\sqrt{k_{c}}$ into Equation (7), we obtain the following:

ΔRX(k_c_)/Δk_c_ ≈ ΔΦ(k_c_)/Δk_c_. (10)

This corresponds to a constant increase in the cross-sectional area of the cylinders: when Δk_c_ = const., then ΔS_c_ = S_0_Δk_c_ = const., as shown in Equation (2).

Analysis of the calculated data curves RX(r_c_) and RΧ(r_sin_) of low- to medium-density PU foams (85 kg/m^3^, 144 kg/m^3^, and 415 kg/m^3^) identified a relationship between the relative partial susceptibilities of corresponding cylinders and shells:

RX(r_c_) + RX(r_sin_) = 1.0. (11)

Then, taking into account r_sin_ = r_c_ for the corresponding cylinders and shells, the model function for shells Ψ(r_sin_) ≈ RX(r_sin_) can be constructed as follows:

(12) $$f_{3}(r_{\sin})=1.0-f_{1}(r_{\sin})=1.0-\frac{1}{\sqrt{2\pi }\sigma_{1}}\int_{0}^{r_{sin}}e^{-\frac{{\left(r-\mu_{1}\right)}^{2}}{2\sigma_{1}^{2}}}dr\text{ at }r_{\sin}<r_{\mathrm{infl}}\text{ and}\;f_{4}(r_{\sin})=1.0-f_{2}(r_{\sin})=1.0-\frac{1}{\sqrt{2\pi }\sigma_{2}r_{sin}}\int_{r_{sin}}^{R_{0}}e^{-\frac{{\left[ln(r)-\mu_{2}\right]}^{2}}{2\sigma_{2}^{2}}}dr\text{ at }r_{\sin}\geq r_{\mathrm{infl}}.$$

For shells, constant increments of radius Δr_sin_ = const. provide radius-dependent increments of coverage coefficient k_s_ as follows:

(13) $${k_{s}}^{\prime }\approx \frac{\Delta k_{s}}{\Delta r_{sin}}=-\frac{2r_{sin}}{R_{0}^{2}},\;\mathrm{then}\;\Delta k_{s}=-2r_{sin}\frac{∆r_{sin}}{R_{0}^{2}}.$$

Therefore, the change rate of RX has to be estimated at constant increments of the OSA sensor’s coverage with dielectric shells:

ΔRX(k_s_)/Δk_s_ ≈ ΔΨ(k_s_)/Δk_s_. (14)

A constant change in the cross-sectional area of a shell is ensured: when Δk_s_ = const., then ΔS_s_ = S_0_Δk_s_ = const., as shown in Equation (2). The ΔRX(k_s_)/Δk_s_ curves for shells, cut from low- to medium-density PU foams (85 kg/m^3^, 144 kg/m^3^, and 415 kg/m^3^), were calculated from model functions according to Equation (14).

Due to a lack of experimental data on the measured permittivity for shells of the high-density PU materials, the corresponding data curves RΧ(r_sin_) could not be calculated. Consequently, Equation (14) could not be used to calculate the change rate of the relative partial susceptibility for shells, i.e., ΔRX(k_s_)/Δk_s_. As an alternative, with ΔΦ(k_c_)/Δk_c_ known for cylinders, its relation to ΔΨ(k_s_)/Δk_s_ for shells was found. Taking into account RX(r_c_) ≈ Φ(r_c_) and RX(r_sin_) ≈ Ψ(r_sin_), we obtain the following from Equation (11):

Φ(r_c_) + Ψ(r_sin_) = 1.0. (15)

Substituting $r_{c}=R_{0}\sqrt{k_{c}}$ and $r_{sin}=R_{0}\sqrt{{1.0-k}_{s}}$ into Equation (15) leads to the following:

Φ(k_c_) = 1.0 − Ψ(k_s_). (16)

Let us give both sides of Equation (16) a finite increment, ΔΦ(k_c_) = −ΔΨ(k_s_), and then divide by Δk_c_, $\frac{∆\Phi (k_{c})}{∆k_{c}}=-\frac{∆\Psi (k_{s})}{∆k_{c}}$. If the coverage coefficient of a cylinder increases, the coverage coefficient of the corresponding shell decreases for the same amount: Δk_c_ = −Δk_s_. Then $\frac{∆\Phi (k_{c})}{∆k_{c}}=\frac{∆\Psi (k_{s})}{∆k_{s}}$, which permits us to calculate ΔΨ(k_s_)/Δk_s_ when ΔΦ(k_c_)/Δk_c_ is known.

In numerical calculations, the functions f_1_(r_c_) and f_2_(r_c_) were determined as normalised, cumulative standard functions NORMDIST(r_c_, μ_1_, σ_1_, TRUE) and LOGNORM.DIST(r_c_, μ_2_, σ_2_) of the MS EXCEL software v.12 (Microsoft Corporation; Redmond, WA, USA). A transformation of the radial coordinate r_c_′ = r_c_ + r_T_ permitted us to translate the function f_2_(r_c_) for a distance r_T_ to the best-fitting position. The parameters of f_1_(r_c_) and f_2_(r_c_), ensuring the best fitting of the calculated RX data, were determined for each dielectric material. Similar calculations were made for shells.

### 3.3. Increments of Radius at Constant Increments of Coverage Coefficient

To illustrate the distribution of the change rate of the relative partial susceptibility over the radius of the sensor’s zone, covered with a dielectric cylinder, the curves “ΔRX(k_c_)/Δk_c_ − k_c_” were recalculated to such r_c_ values, which correspond to constant increments of the coverage coefficient Δk_c_ = k_c(i+1)_ − k_ci_ = const. Here “k_ci_” and “k_c(i+1)_” are points on the k_c_ − axis, i = 1, …, I + 1 and I = k_c_/Δk_c_ = 1.0/Δk_c_. Taking into account that k_ci_ = (r_ci_/R_0_)^2^, the corresponding increments of the radius of a cylinder were calculated as follows:

(17) $$\Delta r_{\mathrm{ci}}(r_{c})=-r_{ci}\pm \sqrt{r_{ci}^{2}+R_{0}^{2}∆k_{c}},\text{ where i}=1,\;2,\;\ldots ,\;I.$$

Since r_c_ ≥ 0.0 mm for all 0.0 mm ≤ r_c_ ≤ R_0_ mm, the positive square root is chosen. Then, the values of Δr_ci_ depend on r_c_, but in a way that ensures Δk_c_ = const., as in Table 1. Similar calculations were made for the curves “ΔRX(k_s_)/Δk_s_ − k_s_” in the case of shells.

Values of the change rate ΔRX(k_c_)/Δk_c_ were calculated numerically at Δk_c_ = 0.05 (5%) for cylinders of PU materials with significantly differing permittivity.

## 4. Results and Discussion

### 4.1. Dielectric Losses

For PU foams with densities of 95–222 kg/m^3^, dielectric losses were measured as ε″(f) = 0.0022–0.0063 (1 kHz) and ε″(f) = 0.0032–0.0084 (0.1 MHz). For monolithic lab-made polyurethane, ε″(f) = 0.042 at 1 kHz and ε″(f) = 0.088 at 0.1 MHz. The acquired data are in good correspondence with the experimental data in [26]. For the monolithic PU, ε′(f) = 3.42 (1 kHz) and the dielectric loss tangent tgδ = ε″(f)/ε′(f) = 0.012 << 1.0. At higher frequencies of the considered range, f = 10 Hz–0.33 MHz, as in Equation (1), the dielectric losses are even smaller. The loss part of the PU materials, ε″(f), is small compared to the real one, ε′(f). Then $\tilde{\varepsilon }$(jf) ≈ ε′(f), and the real part ε′(f) = ε(f) is referred to as permittivity.

### 4.2. Lateral Dimensions of Complete Samples

For PU foams of densities 50–228 kg/m^3^ and ε_t_ = 1.14–1.42 (1 kHz), the penetration depth was determined as 5.72 mm ≤ t_3%_ ≤ 5.87 mm ± 0.02 mm. That corresponds to the conclusions in [7]:, according to the definition of penetration depth, which is proposed in [7], the penetration depth of the OSA sensor increases as the permittivity of the sample increases. In order to achieve the same 3% difference, samples with high values of the true permittivity ε_t_ need to have a bigger penetration depth, t_3%_, whereas samples with low ε_t_ values can have smaller t_3%_ values to achieve the same percentage of difference [8]. The samples have to be thick enough to provide the true permittivity; therefore, 3–4 times the thickness of the penetration depth t_3%_ was taken as appropriate for PUR foams’ samples with densities of 50–1280 kg/m^3^ and t ≈ 20–25 mm [8]. Due to the limited dimensions of the lab-made monolithic PU rods, the semi-cylindrical samples were made with a thickness of 12 mm.

### 4.3. Transversal Dimensions of Complete Samples

The measured permittivity of PU foams and the expanded uncertainty U95% of the experimental data point are practically equal for all three considered kinds of samples: “(1)”, “(2)”, and “(3)”. The differences lie in the limits of the uncertainty of the spectrometer U_S_95.45% = ±0.01, as shown in Table 2. No correlation was identified between the size/shape of the samples and the measured permittivity.

It is concluded that the measured permittivity ε_m_, determined for parallelepiped-shaped samples, which ensure a 100% coverage of the sensor’s active area, differs insignificantly from the true permittivity value ε_t_, measured for complete cylindrical samples, exactly matching the sensor’s active area. All three kinds of samples can be used to measure the true permittivity of PU foams.

For the parallelepiped-shaped PU foam samples with a concentric cylindrical hole (D_0_ = 45 mm), the readings of the spectrometer were equal to those acquired in measurements with no sample on the sensor (“Sensor in air”): ε ≈ 1.00 ± 0.01. PU foams, located outside the cylindrical zone above the active area of the sensor, have practically no effect on the measured permittivity value.

### 4.4. Inner Vertical Surfaces

For both PU materials, monolithic and cellular, the results at each frequency f_n_ (data at f_1_ = 10 Hz, f_8_ = 1280 Hz, and f_16_ = 327,680 Hz are displayed) showed a small relative difference between the permittivity of a complete cylindrical sample ε_t_ and the measured permittivity of two semi-cylindrical samples ε_mc_: R = (ε_t_ − ε_mc_)/ε_t_; |R| < 0.5%, Table 3.

It was concluded that a similar result could be expected for the lab-made monolithic PU as well. The inner vertical surfaces between the semi-cylinders have a negligible impact on the measured permittivity of a combined sample.

### 4.5. Coverage Coefficients

**Figure 7:**
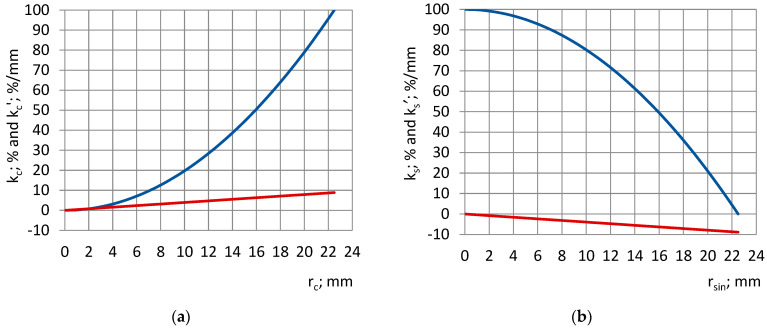
Coverage coefficients k_c_ and k_s_ (blue) and their change rates k′_c_ and k′_s_ (red), depending on (**a**) the radiuses of the cylinders and (**b**) the inner radiuses of the shells.

At an increase in the radius of the cylinders r_c_ and the inner radius of the shells r_sin_ from 0.0 mm to R_0_ = 22.5 mm, the coverage coefficient of the cylinders k_c_ increases nonlinearly from 0% to 100%, while that of shells k_s_ decreases in the same way, as in Figure 7. The change rate k_c_′ increases linearly, and k_s_′ decreases by the same amount.

### 4.6. The Measured Permittivity of Concentric Circular Subsamples

The theoretical parameters of the PU foam SikaBlock-M240 (230 kg/m^3^) sample and subsamples are given in Table 4. The actual dimensions, in Table 5, can differ slightly. Around 5% of the cross-sectional area of a complete sample is changed into sawdust while cutting the three circular lines, each of width w ≈ 0.3 mm. As a result, the actual coverage coefficients k_c_ and k_s_ differ from those calculated with the assumption w = 0.0 mm.

Analysis of experimental data showed that the true permittivity of a complete cylindrical sample can be calculated from the measured permittivity of the four concentric circular subsamples, each of them covering the sensor’s active area partially, as follows:

(18) $$\varepsilon_{t}\approx \varepsilon^{\prime }=\varepsilon_{\mathrm{mc}}+\sum_{j=1}^{3}\varepsilon_{msj}-3.0=1.38.$$

where ε_mc_ and ε_ms_ are the measured permittivities of the subsamples. The relative difference between the true permittivity of the complete cylinder and (1) the calculated permittivity ε′ is estimated as R_1_ = (ε_t_ − ε′)/ε_t_ = (1.40 − 1.38)/1.14 ≈ 1.4% and (2) the measured permittivity of the combined sample ε_m_ as R_2_ = (ε_t_ − ε_m_)/ε_t_ < 1.0%. The true susceptibility χ_t_ of the complete cylindrical sample was determined from the calculated partial susceptibilities of the four concentric circular subsamples as follows:

(19) $$\begin{array}{c} \chi_{\mathrm{pc}}=\varepsilon_{\mathrm{mc}}-1.0;\;\chi_{\mathrm{ps}}=\varepsilon_{\mathrm{ms}}-1.0,\text{ then}\\ \chi_{t}\approx \chi^{\prime }=\chi_{\mathrm{pc}}+\sum_{j=1}^{3}\chi_{psj}+1.0=1.38. \end{array}$$

It can be seen that, although the subsamples have similar coverage coefficients k_c_ ≈ k_s_, the partial susceptibilities differ considerably, as shown in Table 5. The cylindrical subsample provides ~90% of the true susceptibility, while the three shells only ~10%, indicating an uneven distribution of the electric charge over the active area of the sensor. In a general case, for J subsamples (J − 1 shells and a cylinder), the following is calculated:

(20) $$\varepsilon_{t}\approx \varepsilon^{\prime }=\varepsilon_{\mathrm{mc}}+\sum_{j=1}^{J-1}\varepsilon_{msj}-J\;\mathrm{and}\;\chi_{t}\approx \chi^{\prime }=\chi_{\mathrm{pc}}+\sum_{1}^{J-1}\chi_{ps}+1.0.$$

### 4.7. The Measured Permittivity and the Partial Susceptibility at a Partial Coverage

#### 4.7.1. PU Foams with Similar True Permittivity

**Figure 8:**
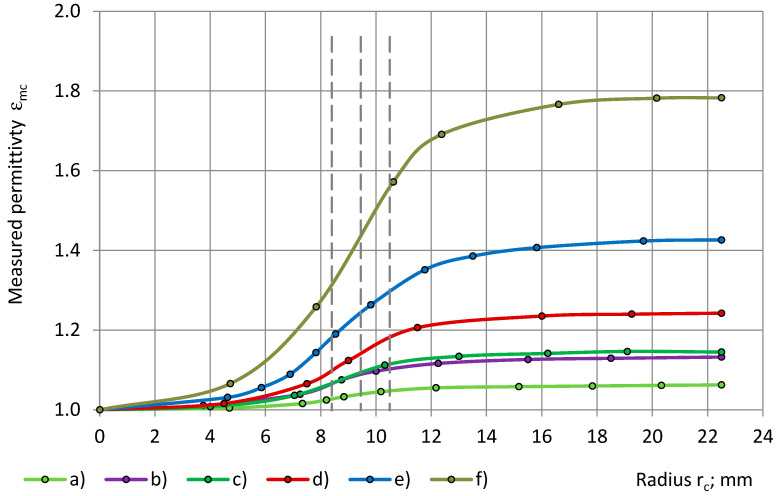
The measured permittivity ε_mc_ with a dependence on the cylinder’s radius r_c_: lab-made PU foams of density (**a**) 32 kg/m^3^ and (**b**) 78 kg/m^3^ and industrial PU foams (**c**) SikaBlock-M80 (85 kg/m^3^), (**d**) SikaBlock-M150 (144 kg/m^3^), (**e**) SikaBlock-M240 (226 kg/m^3^), and (**f**) SikaBlock-M450 (415 kg/m^3^) at f = 1 kHz.

The experimental data of the measured permittivity of low- to medium-density PU foams (32–415 kg/m^3^), with similar true permittivity 1.00 < ε_t_ < 1.80, are depicted in Figure 8 with a dependence on the radius of the cylinders.

At an increase of r_c_ from 0.0 mm to R_0_ = 22.5 mm, the measured permittivity ε_mc_ increases from 0.0 to the value of the true permittivity ε_t_. The concave–onvex curves “ε_mc_ − r_c_” have inflection points, located at the limits of r_e2_ ≤ r_c_ ≤ r_e1in_, where r_e1in_ is the inner radius of electrode №1, and r_e2_ is the radius of electrode №2. The dashed verticals in Figure 8 and other Figures mark the location of the radiuses r_e1in_ and r_e2_ of the sensor’s electrodes №1 and №2 as well as the radius, r_e3_, of the thickness centre of the guard electrode, as in Table 6. Coverage coefficients are given for cylinders in Table 6, when the radius of a cylinder r_c_ = r_e1in_, r_c_ = r_e1out_, r_c_ = r_e2_, and r_c_ = r_e3_, as well as for shells, when the inner radius of a shell r_sin_ = r_e1in_, r_sout_, r_sin_ = r_e2_, and r_sin_ = r_e3_.

The cross-sectional area of a cylinder increases, as its radius increases, from the centre of the cylinder to its outer perimeter. The cross-sectional area of a shell increases as the inner radius of the shell decreases, in the direction from the outer perimeter of the shell to its centre. The ring-shaped area between the electrodes №1 and №2 corresponds to ≈7.9% of the active area (for a cylinder 21.8% − 13.9% = 7.9% as well as for a shell, 86.1% − 78.2% = 7.9%).

**Figure 9:**
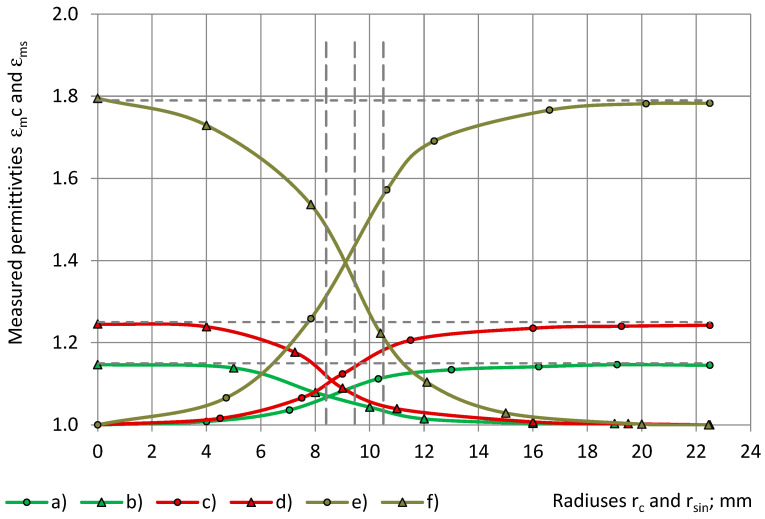
The measured permittivity ε_mc_ of PU foams’ cylinders: (**a**,**c**,**e**) as well as ε_ms_ of shells: (**b**,**d**,**f**), with a dependence on the subsamples’ radiuses r_c_ (circles) and r_sin_ (triangles). Densities: (**a**,**b**) SikaBlock-M80 (85 kg/m^3^), (**c**,**d**) SikaBlock-M150 (144 kg/m^3^), and (**e**,**f**) SikaBlock-M450 (415 kg/m^3^) at f = 1 kHz.

Figure 9 gives experimental data of the measured permittivity ε_mc_ with a dependence on radiuses r_c_ and r_sin_ of the cylinders and shells.

The curves “ε_mc_ − r_c_” and “ε_ms_ − r_sin_ are symmetrical about a straight line, drawn parallel to the Or_c_ (Or_sin_) axis and passing through the intersection point of the curves. Then, for each radius of a cylindrical subsample, 0.0 mm ≤ r_c_ ≤ 22.5 mm, the following relation holds:

ε_c_(r_c_) + ε_s_(r_sin_) = ε_t_. (21)

It can be seen that the true permittivity of PU foams can be determined as a sum of the measured permittivity of the corresponding circular subsamples: a cylinder and a shell. The intersection points of the curves practically coincide with the inflection points.

**Figure 10:**
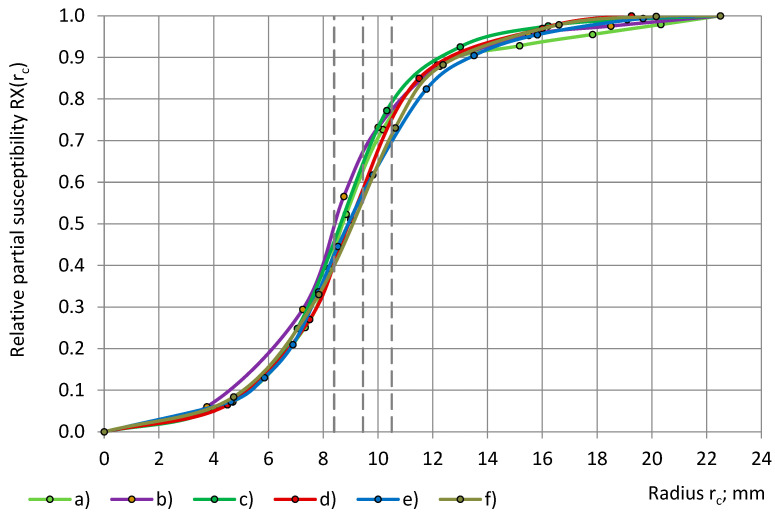
The relative partial susceptibility RX with a dependence on the cylinder’s radius r_c_: lab-made PU foams with densities of (**a**) 32 kg/m^3^ and (**b**) 78 kg/m^3^ and industrial PU foams (**c**) SikaBlock-M80 (85 kg/m^3^), (**d**) SikaBlock-M150 (144 kg/m^3^), (**e**) SikaBlock-M240 (226 kg/m^3^), and (**f**) SikaBlock-M450 (415 kg/m^3^) at f = 1 kHz.

**Figure 11:**
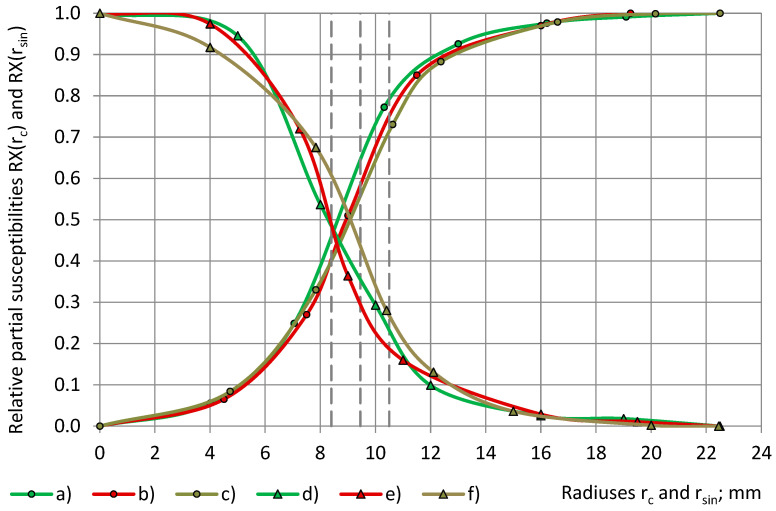
The relative partial susceptibility RX of PU foams’ cylinders (**a**,**c**,**e**) as well as of the corresponding shells (**b**,**d**,**f**) with a dependence on the subsamples’ radiuses r_c_ (circles) and r_sin_ (triangles). Densities: (**a**,**d**) SikaBlock-M80 (85 kg/m^3^), (**b**,**e**) SikaBlock-M150 (144 kg/m^3^), and (**c**,**f**) SikaBlock-M450 (415 kg/m^3^) at f = 1 kHz.

The transition to the calculated relative partial susceptibility RX, as in Equations (6), brings the experimental data from Figure 8 and Figure 9 to a common scale of 0.0 ≤ RΧ(r_c_) and RΧ(r_sin_) ≤ 1.0, as in Figure 10 and Figure 11.

The parameters of the best-fitting model functions Φ(r_c_) and Ψ(r_sin_) of the calculated relationships “RX(r_c_) − r_c_” and “RX(r_sin_) − r_sin_” are given in Table 7. Theoretically, the parameters of Ψ(r_sin_) have to be the same as those of Φ(r_c_), as in Equations (7) and (10), but practically, they can differ due to irregularities in the subsamples’ shape, density, etc. For PU foams of a similar true permittivity, 1.00 < ε_t_ < 1.80, the curves “RX(r_c_) − r_c_” and “RX(r_sin_) − r_sin_” have a similar shape, are close together, and partially overlap, as in Figure 10 and Figure 11, which hinders the determination of the parameters and the identification of the main trends.

**Figure 12:**
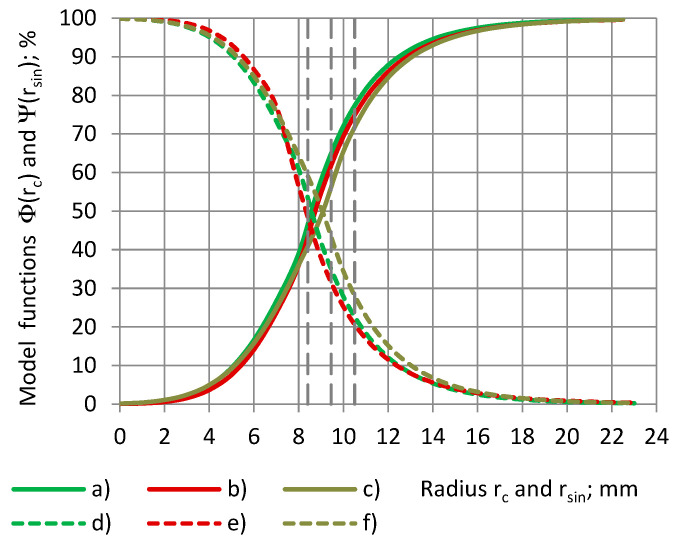
Model functions Φ and Ψ of the relative partial susceptibility RX of PU foams’ cylinders (**a**–**c**) as well as of the corresponding shells (**d**–**f**) with a dependence on the radiuses of subsamples r_c_ and r_sin_. Densities: (**a**,**d**) SikaBlock-M80 (85 kg/m^3^), (**b**,**e**) SikaBlock-M150 (144 kg/m^3^), and (**c**,**f**) SikaBlock-M450 (415 kg/m^3^) at f = 1 kHz.

It can be seen that the model curves “Φ(r_c_) − r_c_” and “Ψ(r_sin_) − r_sin_” for cylinders and shells of both PU foams are symmetrical about a straight line, which is drawn parallel to the Or_c_ (Or_sin_) axis and passes through the intersection of the curves at Φ(r_c_) = Ψ(r_sin_) = 50%, as in Figure 12. Then, for each radius of a cylinder, 0.0 mm ≤ r_c_ ≤ 22.5 mm, and the radius of the cylinder’s corresponding shell, r_sin_ = r_c_, the following hold:

Φ(r_c_) + Ψ(r_sin_) = 1.0 and RX(r_c_) + RX(r_sin_) = 1.0. (22)

**Figure 13:**
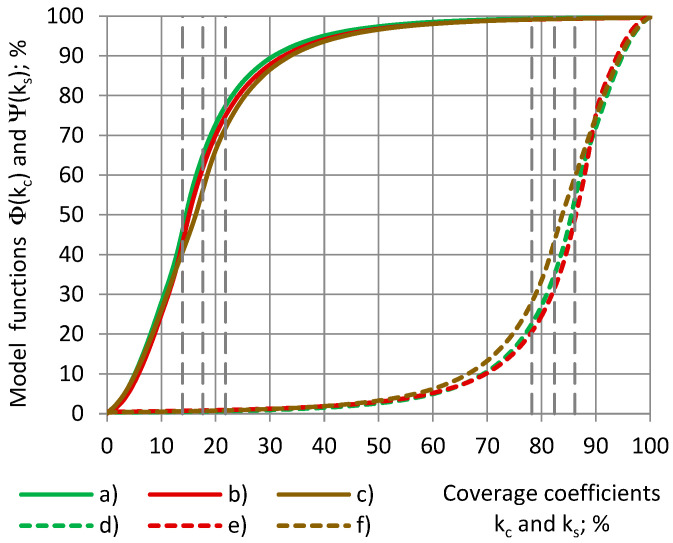
Model functions Φ and Ψ of the relative partial susceptibility RX of PU foams’ cylinders (**a**–**c**) as well as of the corresponding shells (**d**–**f**) with a dependence on the coverage coefficients k_c_ and k_s_. Densities: (**a**,**d**) SikaBlock-M80 (85 kg/m^3^), (**b**,**e**) SikaBlock-M150 (144 kg/m^3^), and (**c**,**f**) SikaBlock-M450 (415 kg/m^3^) at f = 1 kHz.

For each cylinder of a coverage coefficient k_c_ 0.0% ≤ k_c_ ≤ 100% and the cylinder’s corresponding shell of a coverage coefficient k_s_, k_c_ + k_s_ = 100% is valid, as shown in Equation (3). Then, from Figure 13 we obtain the following:

Φ(k_c_) + Ψ(k_s_) = Φ(k_c_) + Ψ(100% − k_c_) = 100%. (23)

#### 4.7.2. PU Materials with Significantly Differing True Permittivity

**Figure 14:**
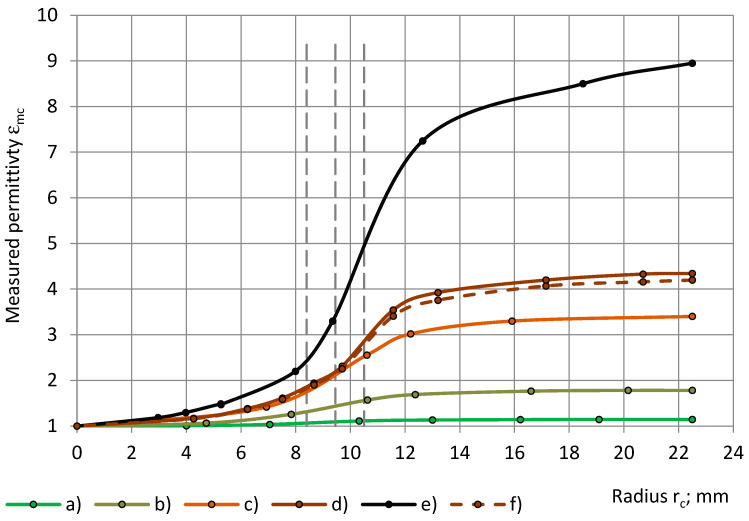
The measured permittivity ε_mc_ with a dependence on the cylinder’s radius r_c_: PU foams (**a**) SikaBlock-M80 (85 kg/m^3^), (**b**) SikaBlock-M450 (415 kg/m^3^), monolithic materials, (**c**) lab-made PU (1280 kg/m^3^), (**d**) industrial PU SikaBlock-M945 (1352 kg/m^3^), (**e**) industrial epoxy Lab-975 New (708 kg/m^3^) at f = 1 kHz, and (**f**) industrial PU SikaBlock-M945 (1352 kg/m^3^) at f = 40960 Hz.

The experimental data of the measured permittivity of dielectrics (PU foams, monolithic polyurethane, and an epoxy) with significantly differing true permittivity (1.0 < ε_t_ < 9.0) are given in Figure 14 with a dependence on the radius of the cylinders. With an increase of r_c_ from 0.0 mm to R_0_ = 22.5 mm, the measured permittivity ε_mc_ increases from 0 to ε_t_.

**Figure 15:**
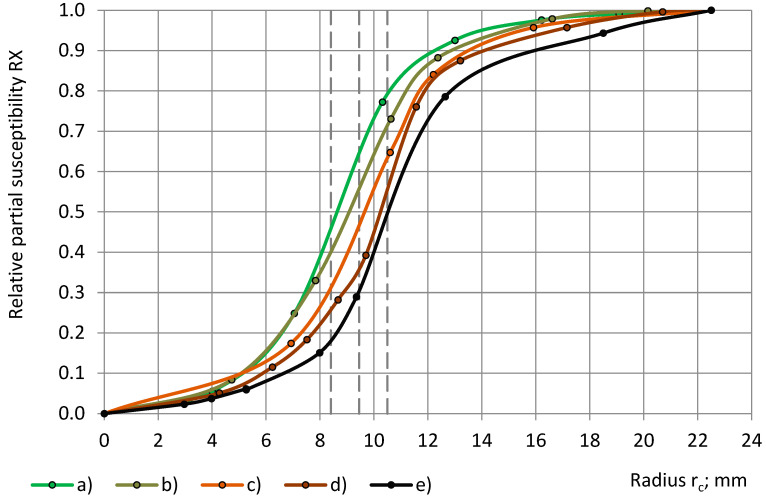
The relative partial susceptibility RX with a dependence on the cylinder’s radius r_c_: (**a**) SikaBlock-M80 (85 kg/m^3^), (**b**) PU foam SikaBlock-M450 (415 kg/m^3^), monolithic materials, (**c**) lab-made PU (1280 kg/m^3^), (**d**) industrial PU SikaBlock-M945 (1352 kg/m^3^), and (**e**) industrial epoxy Lab-975 New (708 kg/m^3^) at f = 1 kHz.

The ε_m_ = ε_m_(r_c_) curves, registered for the light-weight PU foam SikaBlock-M80 (85 kg/m^3^) at frequencies 1000 Hz and 40,960 Hz, practically overlap, because the differences Δε_m_ between the ε_m_ values at both frequencies are less than 0.001. For the monolithic polyurethane SikaBlock-M945 (1352 kg/m^3^), the ε_m_ = ε_m_(r_c_) curve at 40,960 Hz is given in Figure 14f and Δε_m_ < 0.20. When the ε_m_ = ε_m_(r_c_) curves are recalculated to the relative partial susceptibility RX, as in Equations (5) and (6), the RX = RX(r_c_) curves at 1000 Hz and 40960 Hz coincide closely for both PU materials and provide no new information. Therefore, the curves RX = RX(r_c_) at f_13_ = 40,960 Hz are not depicted in Figure 15. It is reasonable to expect the same for PU foams of density values below the density of monolithic PU SikaBlock-M945. It is concluded that the studied PU materials exhibit low dielectric dispersion.

The calculated data curves of the relative partial susceptibility RX(r_c_) follow a similar, concave/convex pattern, with inflection points at the limits, r_e2_ ≤ r_c_ ≤ r_e1in_, as in Figure 15. For dielectrics with higher true permittivity, the inflection points correspond to higher values of the radius of the cylinders, r_c_.

The ε_t_ values of the considered dielectrics are sufficiently different to prevent overlapping of the curves RX(r_c_) and to facilitate a determination of the main trends and parameters of model functions, as shown in Table 8.

**Figure 16:**
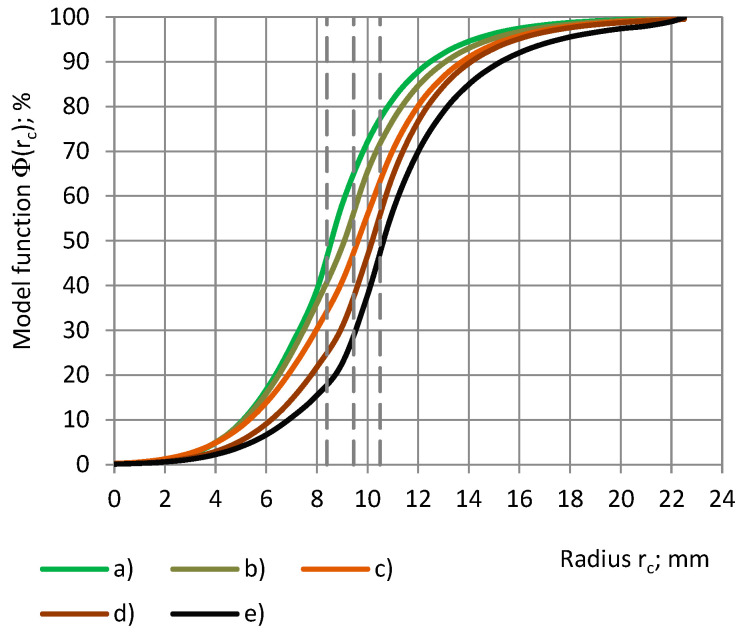
Model functions Φ of the relative partial susceptibility RX with a dependence on the cylinder’s radius r_c_: (**a**) SikaBlock-M80 (85 kg/m^3^), (**b**) PU foam SikaBlock-M450 (415 kg/m^3^), monolithic materials, (**c**) lab-made PU (1280 kg/m^3^), (**d**) industrial PU SikaBlock-M945 (1352 kg/m^3^), and (**e**) an epoxy Lab-975 New (708 kg/m^3^) at f = 1 kHz.

The main features of the calculated data curves RX(r_c_), as in Figure 15, are valid for the model functions Φ(r_c_) of the relative partial susceptibility as well, as in Figure 16. Compared to the PU foam SikaBlock-M80 (ε_t_ = 1.15), the inflection point of the Φ = Φ(r_c_) curve for epoxy LAB975 (ε_t_ = 8.95) is located ≈2 mm further in the direction of the higher values of r_c_. This can be explained by a higher input of the outer circular zones (covered with the dielectric) of the electrode №1 into the total transcapacitance. At higher values of the true permittivity, the input becomes sufficient to be detected by a spectrometer of a certain sensitivity.

For PU foams with densities of 78–85 kg/m^3^, the true permittivity 1.13 ≤ ε_t_ ≤ 1.15 is only ≈13–15% higher than that of vacuum ε_t0_ = 1.0. The outer circular zones of the electrode №1 (r_c_ > 12 … 15 mm) have a lower charge density, compared to that of the inner zones [2]; as a result, their input is comparatively small.

**Figure 17:**
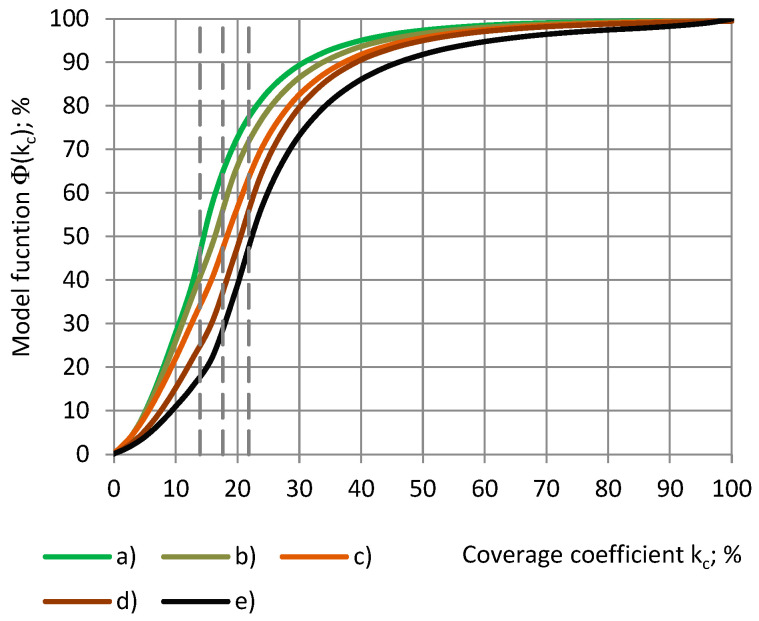
Model functions Φ of the relative partial susceptibility RX with a dependence on the coverage coefficient k_c_ of the cylinders: (**a**) SikaBlock-M80 (85 kg/m^3^), (**b**) PU foam SikaBlock-M450 (415 kg/m^3^), monolithic materials, (**c**) lab-made PU (1280 kg/m^3^), (**d**) industrial PU SikaBlock-M945 (1352 kg/m^3^), and (**e**) an epoxy Lab-975 New (708 kg/m^3^) at f = 1 kHz.

The higher the true permittivity, the higher the coverage coefficient, at which a certain value of the relative partial susceptibility RX is reached: (a) 50%, (b) 75%, and (c) 90%, as in Figure 17.

**Figure 18:**
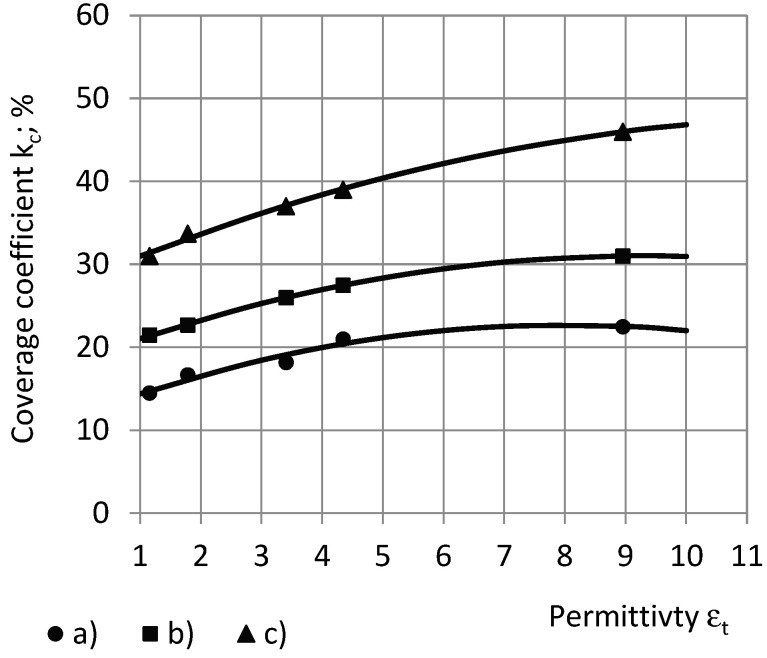
The dependence of the coverage coefficient k_c_ on the true permittivity ε_t_ at the relative partial susceptibility RX (**a**) 50%, (**b**) 75%, and (**c**) 90%.

The mentioned trend is displayed in Figure 18.

This may be explained by a higher input of the outer circular zones of electrode №1 into the total transcapacitance, when at a higher true permittivity, the input becomes sufficient to be detected by a spectrometer of a certain sensitivity.

The coverage of the sensor’s active area increases from 0% to 100% in the direction from (1) the centre to the perimeter at an increase of a cylinder’s radius from 0 to R_0_, and when Δr_c_ > 0, then Δk_c_ > 0, and in the direction from (2) the perimeter to the centre at a decrease of a shell’s inner radius from R_0_ to 0, and when Δr_sin_ < 0, then Δk_s_ > 0. The values of the change rate ΔRX(k_c_)/Δk_c_ were calculated numerically at Δk_c_ = Δk_s_ = 0.05 (5%).

**Figure 19:**
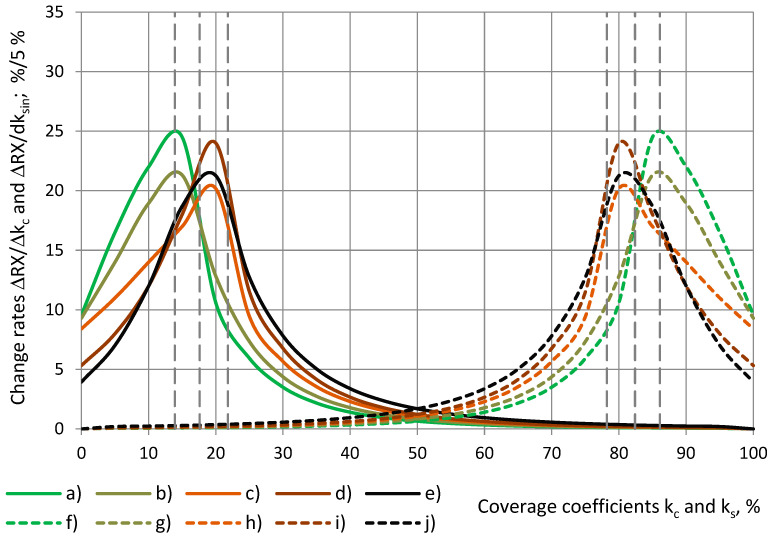
The change rate of the relative partial susceptibility, with a dependence on the coverage coefficient of the cylinders (continuous lines) and of shells (dashed lines): (**a**,**f**) SikaBlock-M80 (85 kg/m^3^), (**b**,**g**) PU foam SikaBlock-M450 (415 kg/m^3^), monolithic materials (**c**,**h**) lab-made PU (1280 kg/m^3^), (**d**,**i**) industrial PU SikaBlock-M945 (1352 kg/m^3^) as well as (**e**,**j**) an epoxy Lab-975 New (708 kg/m^3^) at f = 1 kHz.

In the centre of the OSA sensor, in Figure 19, at k_c_ = 0% for cylinders, the change rate is ΔRX/Δk_c_ ≈ 4–10%/5%, because the change rate value ΔRX/Δk_c_, calculated for each coverage interval Δk_c_ = k_c(i+1)_ − k_ci_ = 5%, is assigned to its left point k_ci_. ΔRX/Δk_c_ has the highest values of 20–25%/5% at 14% < k_c_ < 22%, which corresponds to the zone between electrodes №1 and №2. In the centre of the sensor, ΔRX/Δk_c_ is 2–5 times smaller, and it decreases to < 5%/5% as k_c_ increases above 35%. For shells, in the centre of the OSA sensor at k_s_ = 100%, the corresponding value of the change rate ΔRX/Δk_s_ ≈ 4–10%/5% is assigned to the right point of each Δk_s_ interval. ΔRX/Δk_s_ has the highest values of 20–25%/5% at 78% < k_s_ < 86% and decreases to less than 5%/5% at k_s_ < 65%, as shown in Table 6.

**Figure 20:**
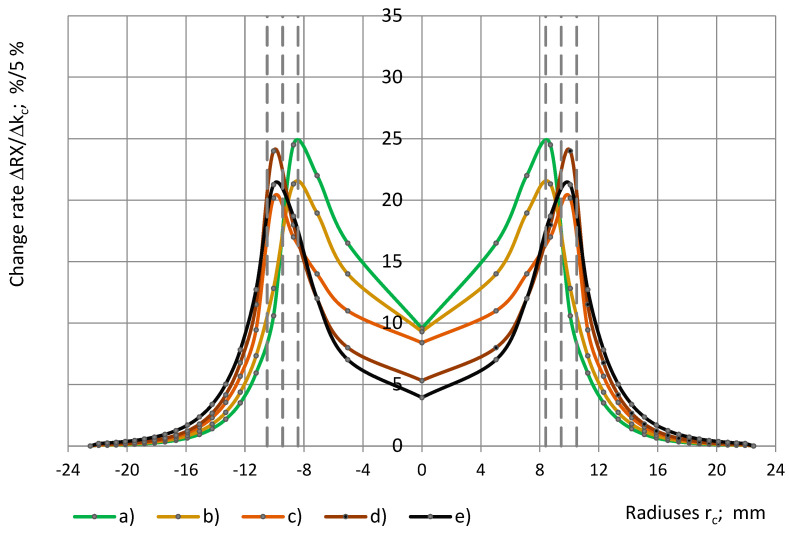
The change rate of the relative partial susceptibility ΔRX/Δk_c_ with a dependence on the radius r_c_ of a cylinder: (**a**) SikaBlock-M80 (85 kg/m^3^), (**b**) PU foam SikaBlock-M450 (415 kg/m^3^), monolithic materials, (**c**) lab-made PU (1280 kg/m^3^), (**d**) industrial PU SikaBlock-M945 (1352 kg/m^3^), as well as (**e**) an epoxy Lab-975 New (708 kg/m^3^) at f = 1 kHz.

Figure 20 shows the change rate of the relative partial susceptibility ΔRX/Δk_c_ over the radius of the sensor’s circular concentric zone, which is covered with a dielectric cylinder of radius r_c_. The increments Δr_ci_, corresponding to the markers on the curves, depend on r_c_ in a way that ensures constant increments of the coverage coefficient Δk_c_ = 5%, as in Equation (17). It can be seen that the change rate is the highest, ΔRX/Δk_c_ = 20–25%/5%, in the ring-shaped zone of radius 8.4 mm < r_c_ < 10.5 mm between electrodes №1 and №2. In the centre of the sensor, the change rate is 2–5 times smaller. As the radius r_c_ increases above 12 mm, ΔRX/Δk_c_ decreases to <5%/5%.

The numerical results of several mathematical models of other authors are available for circular OSA sensors with two electrodes in [2,7,13,15]. The overall character of the curves in Figure 20 like slopes of lines and the ratio of ΔRX/Δk_c_ values in the centre and near the gap corresponds with the character of the surface charge density distribution curves, calculated from mathematical modelling. In [11], a simplification is made by assuming an even distribution of the surface charge on the sensor’s electrodes. In [12,13], a complex mathematical model for a circular three-electrode OSA sensor is elaborated, but numerical results for the surface charge are not displayed.

A shortage of experimental data for the surface charge density distribution over the active area of circular OSA sensors hinders a relevant comparison.

## 5. Conclusions

The electric susceptibility at the partial coverage of a circular OSA sensor with cylinders and shells is investigated for PU foams and monolithic polyurethane. It is shown experimentally that the true susceptibility can be determined from the partial susceptibilities of the corresponding subsamples. The implementation of the relative partial susceptibility permitted us to transform the calculated susceptibility data to a common scale of 0.0–1.0 and to outline the main trends for PU materials.

The partial susceptibility, the relative partial susceptibility, and the change rate of the relative partial susceptibility exhibit a marked dependence on the coverage coefficient of the OSA sensor’s active area as well as a correlation with the coordinates of the sensor’s electrodes and the gap. Numerical calculations showed that, for cylindrical subsamples, the change rate of the relative partial susceptibility is the highest in the zone of the active area between electrodes №1 and №2: 20–25%/5%. In the centre of the sensor, it is 2–5 times smaller and decreases below 5%/5% as the coverage coefficient increases above 35% in correspondence with the surface charge density distribution, reported in scientific information sources. The curves for shells are symmetric to those for the cylinders with respect to the location of the gap. The overall pattern of curves for the change rate of the relative partial susceptibility, characterised by slopes of lines and the ratio of ΔRX/Δk_c_ values in the centre and values near the gap, corresponds with the character of the surface charge density distribution curves, calculated from mathematical models.

The uncertainty of the spectrometer, geometric irregularities of subsamples, and varying humidity of the ambient atmosphere can be named as the main sources of measurement uncertainties for PU foams. The elaborated methods can be applied in the design and optimization of capacitive OSA sensors of other configurations of electrodes, independent of the particular technical solution.

## Figures and Tables

**Table 1:** Increments of cylinder’s radius Δr_c_ at a constant increment of the coverage coefficient Δk_c_ = 5%.

i	Coverage Coefficient k_c_	Radius r_c_; mm	Increment Δr_c_; mm	i	Coverage Coefficient k_c_	Radius r_c_; mm	Increment Δr_c_; mm
1	0.00	0.0	5.03	12	0.55	16.7	0.74
2	0.05	5.0	2.08	13	0.60	17.4	0.71
3	0.10	7.1	1.60	14	0.65	18.1	0.68
4	0.15	8.7	1.35	15	0.70	18.8	0.66
5	0.20	10.1	1.19	16	0.75	19.5	0.64
6	0.25	11.3	1.07	17	0.80	20.1	0.62
7	0.30	12.3	0.99	18	0.85	20.7	0.60
8	0.35	13.3	0.92	19	0.90	21.3	0.58
9	0.40	14.2	0.86	20	0.95	21.9	0.57
10	0.45	15.1	0.82	21	1.00	22.5	0.00
11	0.50	15.9	0.78				

**Table 2:** The measured permittivity and measurement uncertainties of PU foams.

N	Density ρ; kg/m^3^	Kind of a Sample	Measured Permittivity ε_m_			Expanded Uncertainty U95%		
			f_1_	f_7_	f_16_	f_1_	f_7_	f_16_
1	50	(1)	1.09	1.09	1.08	0.010	0.010	0.010
		(2)	1.10	1.09	1.08	0.010	0.010	0.010
		(3)	1.09	1.09	1.08	0.010	0.010	0.010
2	144	(1)	1.28	1.26	1.24	0.013	0.010	0.100
		(2)	1.27	1.26	1.25	0.012	0.010	0.100
		(3)	1.27	1.26	1.24	0.012	0.010	0.100
3	427	(1)	1.86	1.84	1.75	0.011	0.011	0.011
		(2)	1.87	1.84	1.76	0.015	0.011	0.010
		(3)	1.86	1.84	1.76	0.011	0.011	0.010

**Table 3:** The permittivity of cylindrical and semi-cylindrical samples.

N	Dielectric Material	Density ρ; kg/m^3^	Sample	Thickness t; mm	Permittivity ε_t_ and ε_mc_		
					f_1_	f_8_	f_16_
1	PU foam SikaBlock-M450	437	A cylinder	12	1.855	1.802	1.756
2			Two semi-cylinders		1.852	1.804	1.757
|R|; %					0.16	0.11	0.06
3	Monolithic PU SikaBlock- M960	1180	A cylinder	12	3.748	3.672	3.507
4			Two semi-cylinders		3.764	3.681	3.520
|R|; %					0.43	0.25	0.37
5	Monolithic PU, lab-made	1280	Two semi-cylinders	12	3.474	3.408	3.310

**Table 4:** Theoretical parameters of the sample and subsamples (d_in_ and d_out_—the inner and outer diameters).

Sample/Subsample	Diameter			Thickness of a Shell Wall t; mm		Cross-Sectional Area S; mm^2^	Coverage Coefficients k_c_ and k_s_; %
	d; mm	d_in_; mm	d_out_; mm				
Cylinder, complete	45.0	-	-	-		1590.4	100
Subsamples							
Cylinder	22.5	0.0	22.5	-		397.6	25
Shell 1	-	22.5	31.8	4.7		397.6	25
Shell 2	-	31.8	39.0	3.6		397.6	25
Shell 3	-	39.0	45.0	3.0		397.6	25
Sum:						1590.4	100
The combined sample							
Cylinder + shell 1 + shell 2 + shell 3	45.0	-	-			1590.4	100

**Table 5:** The parameters of the sample and subsamples, determined experimentally.

Sample/Subsample	Diameter			Thickness of a Shell Wall t; mm	Area S; mm^2^	k_c_ and k_s_; %	ε_t_ and ε_m_(1 kHz)	Susceptibility χ_t_ and χ_p_ (1 kHz)	χ_p_/Sumχ_p_; %
	d; mm	d_in_; mm	d_out_; mm						
Cylinder, complete	44.9	-	-	-	1583.4	99.6	1.40	0.40	
Subsamples									
Cylinder	22.2	-	-	-	387.1	24.3	1.34	0.34	89.3
Shell 1	-	22.8	31.5	4.35	371.0	23.3	1.03	0.03	8.1
Shell 2	-	32.1	38.7	3.3	367.0	23.1	1.01	0.01	2.1
Shell 3	-	39.1	44.9	2.9	382.6	24.1	1.00	0.00	0.5
Sum:					1507.7	94.8	4.38	0.38	100.0
The combined sample									
Cylinder + shell 1 + shell 2 + shell 3	44.9	-	-	-	1507.7	94.8	1.40	0.40	100.0

**Table 6:** Radiuses of the sensor’s electrodes and corresponding coverage coefficients for cylinders and shells.

Radius r_e1in_; mm	Coverage Coefficients k_c_ and k_s_; %	Radius r_e1out_; mm	Coverage Coefficients k_c_ and k_s_; %	Radius r_e2_; mm	Coverage Coefficients k_c_ and k_s_; %	Radius r_e3_; mm	Coverage Coefficients k_c_ and k_s_; %
10.50	21.8	22.50	100.0	8.40	13.9	9.45	17.6
	78.2		0.0		86.1		82.4

**Table 7:** Parameters of the model functions Φ(r_c_) and Ψ(r_sin_) for cylinders and shells.

N	PU Foams	Density ρ; kg/m^3^	True Permitt. ε_t_ (1 kHz)	Model Functions Φ(r_c_) and Ψ(r_sin_)					
				Normal Cumulative f_1c_(r_c_) or f_1s_(r_sin_)			Lognormal Cumulative f_2c_(r_c_) or f_2s_(r_sin_)		
				μ_1_	σ_1_	Logical	μ_2_	σ_2_	r_T_
				Cylinders					
1	SikaBlock-M80	85	1.15	8.8	2.9	TRUE	1.32	0.56	−4.8
2	SikaBlock-M150	144	1.24	9.0	2.8	---“---	1.29	0.59	−5.1
3	SikaBlock-M450	415	1.78	9.1	3.1	---“---	1.32	0.57	−5.3
				Shells					
1	SikaBlock-M80	85	1.15	8.8	2.9	TRUE	1.32	0.56	−4.8
2	SikaBlock-M150	144	1.24	9.0	2.7	---“---	1.10	0.67	−5.3
3	SikaBlock-M450	415	1.78	9.1	3.1	---“---	1.32	0.57	−5.3

**Table 8:** The parameters of model function Φ(r_c_) (cylinders).

N	Dielectric Material	Density; kg/m^3^	Permitt. ε_t_ (1 kHz)	Model Function Φ(r_c_)					
				Normal Cumulative f_1_(r_c_)			Lognormal Cumulative f_2_(r_c_)		
				μ_1_	σ_1_	Logical	μ_2_	σ_2_	r_T_
1	Monolithic PU; lab-made	1280	3.40	9.8	3.5	TRUE	1.30	0.58	−6.0
2	Monolithic PU Sika M945	1352	4.34	10.8	3.6	---“---	1.15	0.63	−7.0
3	Epoxy LAB975	708	8.95	12.0	4.0	---“---	1.10	0.73	−7.6

## Data Availability

Data are contained within this article.
